# The association of apolipoproteins with later-life all-cause and cardiovascular mortality: a population-based study stratified by age

**DOI:** 10.1038/s41598-021-03959-5

**Published:** 2021-12-24

**Authors:** Mozhu Ding, Alexandra Wennberg, Stina Ek, Giola Santoni, Bruna Gigante, Göran Walldius, Niklas Hammar, Karin Modig

**Affiliations:** 1 Unit of Epidemiology, Institute of Environmental Medicine, Karolinska Institutet, Nobelsväg 13, 17177 Stockholm, Sweden; 2 Upper Gastrointestinal Surgery, Department of Molecular Medicine and Surgery, Karolinska Institutet, Karolinska University Hospital, Stockholm, Sweden; 3 Division of Cardiovascular Medicine, Department of Medicine, Karolinska Institutet, Stockholm, Sweden; 4 Division of Cardiology, Danderyd University Hospital, Stockholm, Sweden

**Keywords:** Predictive markers, Risk factors, Cardiovascular diseases, Epidemiology

## Abstract

Midlife lipid levels are important predictors of cardiovascular diseases, yet their association with mortality in older adults is less clear. We aimed to (1) identify lipid profiles based on cholesterol, triglycerides, and apolipoproteins using cluster analysis, and (2) investigate how lipid profiles and lipid levels at different ages are associated with later-life all-cause and cardiovascular mortality. We used data from 98,270 individuals in the Swedish AMORIS cohort who had blood measurements between 1985–1996 and were followed until 2012. Over the follow-up (mean 18.0 years), 30,730 (31.3%) individuals died. Three lipid profiles were identified. Compared with reference profile, a high lipid profile (low ApoA-I and high total cholesterol (TC), triglycerides, ApoB, and ApoB/ApoA-I ratio) at ages 39–59 or 60–79 was associated with higher all-cause mortality. A high lipid profile at ≥ 80 years, however, did not confer higher mortality. For the specific markers, high TC (≥ 7.25 mmol/L) was associated with higher all-cause mortality in ages 39–59 but lower mortality in ages 60–79 and ≥ 80. Low ApoA-I (< 1.28 g/L) and high ApoB/ApoA-I ratio (≥ 1.18), on the other hand, were associated with higher cardiovascular mortality regardless of age at lipid measurement, highlighting their potential relevance for survival in both young and older individuals.

## Introduction

Elevated lipid levels are important predictors of cardiovascular morbidity and mortality in middle-aged populations^1,2^. In older adults, however, evidence on the association between high cholesterols and health outcomes is conflicting, with some studies reporting a null or reversed association between high total cholesterol (TC) or low-density lipoprotein cholesterol (LDL-C) levels and all-cause mortality^3–6^. It was therefore proposed that for people who survived to advanced ages high cholesterol levels may no longer be a risk factor for mortality. Yet current evidence is inconclusive as previous studies differ substantially in sample size and follow-up time and few had enough power to perform meaningful age-stratified analysis. In addition, the role of lipid biomarkers other than cholesterols for mortality in old age is unclear. For instance, apolipoprotein B (ApoB), ApoA-I, and ApoB/ApoA-I ratio have been shown to have better prediction of cardiovascular outcomes than cholesterol^2,7^, but whether these markers measured in older ages still predict mortality is not known.

Moreover, there is a knowledge gap regarding how lipid biomarkers operate together to influence mortality. Previous studies have mainly focused on one biomarker at a time^3,8,9^, and do not attempt to capture the heterogeneity of lipid metabolism within the study population. Cluster analysis is a potentially powerful tool to group individuals based on their heterogeneity in an array of lipid biomarkers (e.g., cholesterols, triglycerides, and apolipoproteins). Therefore, multiple co-occurring lipid levels can be captured in a cluster, making it a promising approach to identify individuals with distinct lipid profiles in the general population who are at different risk of mortality.

Therefore, the aim of this study is to (1) identify distinct lipid profiles in a population-based setting based on cholesterol, triglycerides, and apolipoproteins using cluster analysis, (2) analyze how different lipid profiles at different ages are associated with later-life all-cause and cardiovascular mortality, and (3) evaluate the association between single lipid biomarkers, including TC, triglycerides, ApoB, ApoA-I, and ApoB/ApoA-I ratio, and later-life all-cause and cardiovascular mortality in different age groups.

## Results

Baseline characteristics are shown in Table 1. Of the 98,270 participants at baseline, the mean age was 57.1 years (SD 9.7) and 46.8% were female. During the follow-up period (mean 18.0 years, SD 6.0), 30,730 (31.3%) participants died (all causes), of whom 18,135 (59.0%) died from cardiovascular diseases (CVD). The proportion that died during the follow-up was 17% among people aged 39–59 years at blood measurement (mean age at death 67.8 years), 55% among those aged 60–79 years (mean age at death 81.4 years), and 91% among those aged ≥ 80 years (mean age at death 91.4 years).

**Table 1:** Characteristics of the study population.

Characteristics	Total (n = 98,270)	Age at blood measurement 39–59 years (n = 63,780)	Age at blood measurement 60–79 years (n = 32,056)	Age at blood measurement ≥ 80 years (n = 2434)
Age at baseline, mean (SD)	57.1 (9.7)	51.2 (4.8)	66.7 (5.4)	83.1 (3.0)
Female sex, n (%)	45,978 (46.8)	26,961 (42.3)	17,266 (53.9)	1751 (71.9)
**Lipid biomarkers at baseline, mean (SD)**				
TC, mmol/L	6.22 (1.16)	6.14 (1.13)	6.40 (1.18)	6.14 (1.22)
Triglycerides, mmol/L	1.43 (0.81)	1.40 (0.82)	1.49 (0.79)	1.41 (0.75)
ApoB, g/L	1.34 (0.34)	1.33 (0.34)	1.37 (0.35)	1.26 (0.34)
ApoA-I, g/L	1.47 (0.23)	1.46 (0.23)	1.48 (0.34)	1.49 (0.35)
ApoB/ApoA-I ratio	0.94 (0.28)	0.94 (0.29)	0.95 (0.28)	0.87 (0.26)
History of CVD at baseline, n (%)	9319 (9.5)	3159 (5.0)	5457 (17.0)	703 (28.9)
Coronary heart disease	4707 (7.8)	1384 (2.2)	2940 (9.2)	383 (15.7)
Heart failure	1299 (1.3)	176 (0.3)	800 (2.5)	253 (10.4)
Atrial fibrillation	1372 (1.4)	348 (0.6)	858 (2.7)	166 (6.8)
Ischemic stroke/TIA	1488 (1.51)	351 (0.6)	985 (3.1)	152 (6.2)
Hypertension	2553 (2.6)	952 (1.5)	1449 (4.5)	152 (6.2)
Diabetes	1552 (1.6)	588 (0.9)	858 (2.7)	106 (4.4)
Follow-up time, mean (SD)	18.0 (6.0)	19.8 (5.0)	15.4 (6.3)	7.8 (4.7)
Age at death, mean (SD)	77.3 (10.3)	67.8 (7.5)	81.4 (7.1)	91.4 (4.6)
Deaths (all causes) during follow-up, n (%)	30,730 (31.3)	10,863 (17.0)	17,650 (55.1)	2217 (91.1)
Cardiovascular deaths during follow-up, n (%)	18,135 (18.5)	5234 (8.2)	11,313 (35.3)	1588 (65.2)

*SD* standard deviation, *CVD* cardiovascular disease, *TIA* transient ischemic attack, *TC* total cholesterol, *ApoB* apolipoprotein B, *ApoA-I* apolipoprotein A-I.

### Cluster analysis

**Figure 1:**
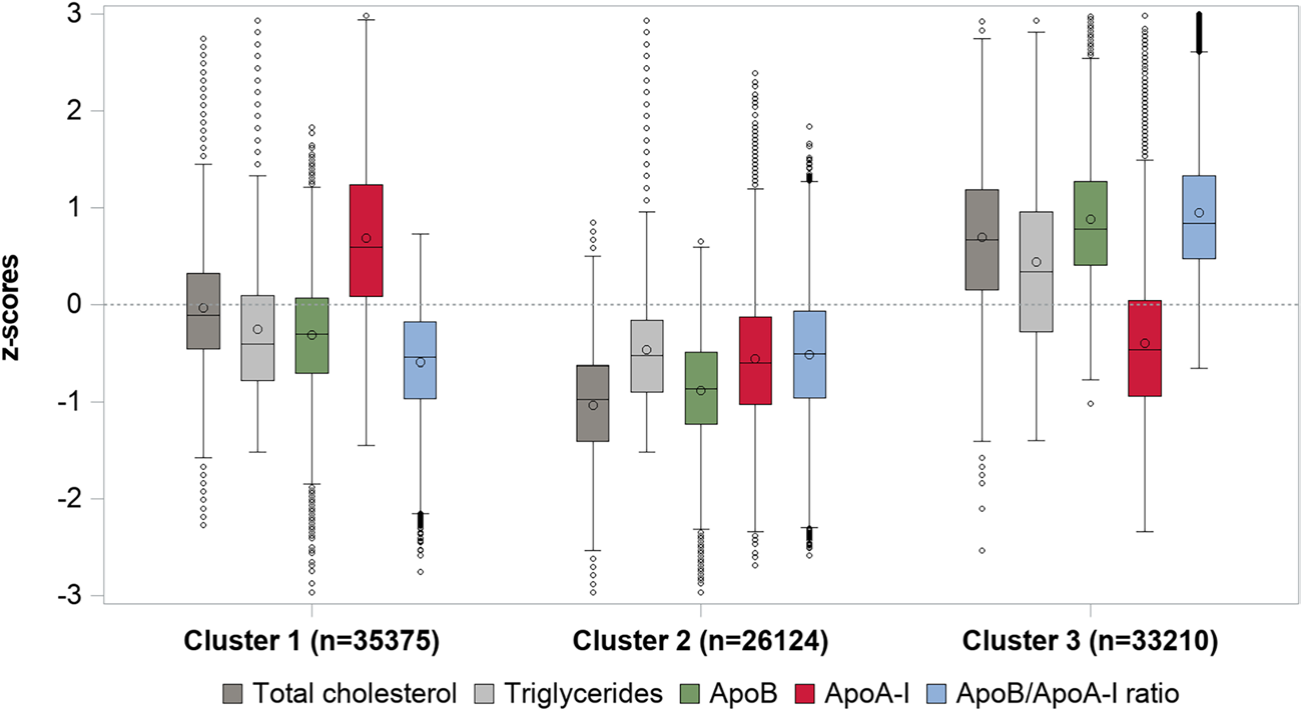
Distribution of total cholesterol, triglycerides, ApoB, ApoA-I, and ApoB/ApoA-I ratio in z-scores in the three identified clusters.

Three clusters were identified, and the distribution of TC, triglycerides, ApoB, ApoA-I, and ApoB/ApoA-I ratio in each cluster are presented in Fig. 1. Cluster 1 included 35,375 individuals and is characterized by a normal TC, normal triglycerides, normal ApoB, high ApoA-I, and low ApoB/ApoA-I ratio, as compared to the sample mean. Cluster 2 included 26,124 individuals and is characterized by low levels on all the studied biomarkers. Cluster 3, including 33,210 individuals, is characterized by a high lipid profile with high TC, triglycerides, ApoB, and ApoB/ApoA-I ratio, and low ApoA-I. There were marked differences in all the biomarkers between the three clusters (Supplementary Figure Table S1 online). Performing the cluster analysis separately in the three age groups produced similar cluster patterns as in the total sample (Supplementary Fig. S2 online).

During the follow-up, both Cluster 2 and Cluster 3 were associated with a higher all-cause and cardiovascular mortality after adjusting for age, sex, and baseline history of CVD (Table 2) when compared with Cluster 1. In the age-specific analyses, Cluster 3 is associated with a hazard ratio (HR) of 1.17 (95% CI 1.11–1.22) for all-cause mortality in age group 39–59 years (i.e., age at blood measurement), an HR of 1.07 (1.03–1.11) in age group 60–79 years, and an HR of 1.01 (0.89–1.15) in age group ≥ 80 years. For cardiovascular mortality, Cluster 3 is associated with an HR of 1.36 (95% CI 1.27–1.45) in age group 39–59 years, an HR of 1.15 (1.09–1.20) in age group 60–79 years, and an HR of 1.05 (0.90–1.23) in age group ≥ 80 years. Cluster 2 was only associated with increased all-cause and cardiovascular mortality in age group 60–79 years, but not in the other two age groups.

**Table 2:** Hazard ratios and 95% confidence interval of all-cause and cardiovascular mortality associated with 3 lipid biomarker clusters in the total sample and by age groups.

	Total sample (n = 94,709)			Age at blood measurement, 39–59 years (n = 61,583)			Age at blood measurement, 60–79 years (n = 30,767)			Age at blood measurement, ≥ 80 years (n = 2359)		
	N^a^	n^b^/1000 PY	HR^c^ (95% CI)	N^a^	n^b^/1000 PY	HR^c^ (95% CI)	N^a^	n^b^/1000 PY	HR^c^ (95% CI)	N^a^	n^b^/1000 PY	HR^c^ (95% CI)
**Outcome: All-cause mortality**												
Cluster 1	35,375	8396/677	Ref (1.00)	26,310	3875/532	Ref (1.00)	8686	4180/142	Ref (1.00)	379	341/106	Ref (1.00)
Cluster 2	26,124	9069/448	1.08 (1.05–1.12)	15,621	2482/306	1.04 (0.99–1.10)	9358	5550/134	1.10 (1.06–1.15)	1145	1037/124	1.09 (0.96–1.23)
Cluster 3	33,210	11,857/586	1.11 (1.07–1.14)	19,652	3938/382	1.17 (1.11–1.22)	12,723	7148/196	1.07 (1.03–1.11)	835	771/113	1.01 (0.89–1.15)
**Outcome: Cardiovascular mortality**												
Cluster 1	35,375	4447/676	Ref (1.00)	26,310	1683/531	Ref (1.00)	8686	2524/142	Ref (1.00)	379	240/74	Ref (1.00)
Cluster 2	26,124	5288/448	1.07 (1.03–1.12)	15,621	1076/306	0.99 (0.92–1.07)	9358	3494/134	1.09 (1.04–1.15)	1145	718/85	1.06 (0.91–1.23)
Cluster 3	33,210	7514/586	1.22 (1.17–1.26)	19,652	2162/382	1.36 (1.27–1.45)	12,723	4775/197	1.15 (1.09–1.20)	835	577/85	1.05 (0.90–1.23)

^a^N = number of subjects. ^b^n = Number of events. ^c^Adjusted for age, sex, and baseline history of coronary heart disease, heart failure, atrial fibrillation, hypertension, diabetes, ischemic stroke, and transient ischemic attack.

*PY* person-years, *HR* hazard ratio, *CI* confidence interval.

### Association between individual lipid biomarkers and mortality

**Figure 2:**
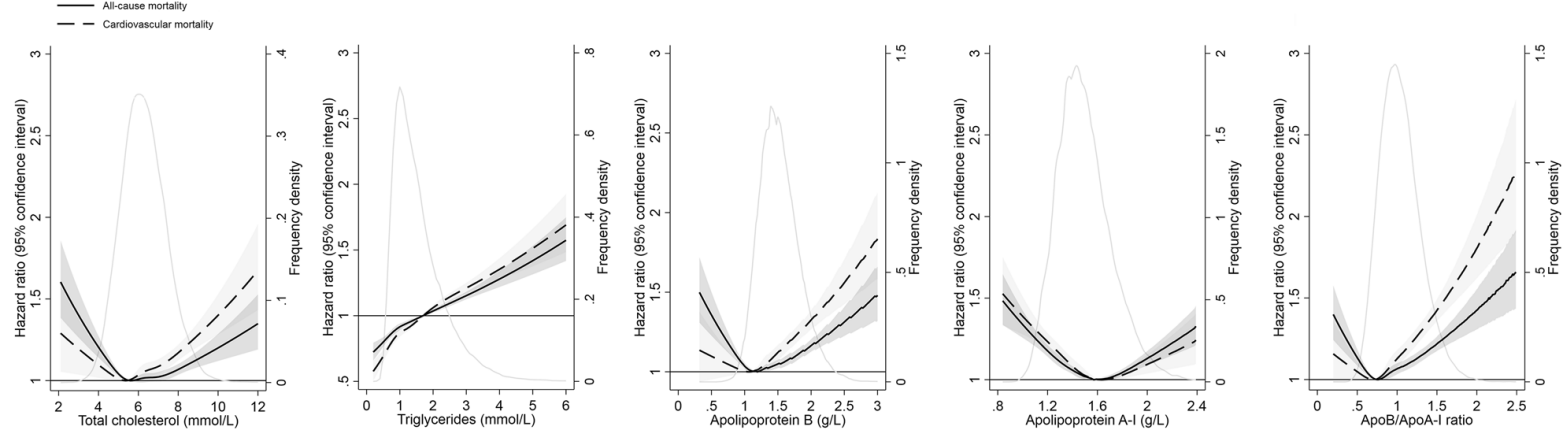
Hazard ratios and 95% confidence interval for all-cause and cardiovascular mortality associated with concentrations of lipid biomarkers on a continuous scale over 25 years of follow-up (n = 98,270). Hazard ratios (solid line for all-cause mortality and dashed line for cardiovascular mortality) and 95% confidence intervals (gray area) are retrieved from Cox regression models with restricted cubic splines, adjusted for age, sex, and history of coronary heart disease, heart failure, atrial fibrillation, hypertension, diabetes, ischemic stroke, and transient ischemic attack. Solid gray line indicates the distribution of lipid biomarkers in the study population.

Figure 2 shows how each lipid biomarker was associated with mortality when the concentrations were analyzed on a continuous scale. TC and ApoA-I showed a U-shaped association with mortality after adjusting for age, sex, and history of CVD, that is, both low and high concentrations were associated with an increased mortality risk. For ApoB and AopB/ApoA-I ratio, the associations were J-shaped, with only slightly elevated risk of mortality for the lowest levels but clearly elevated risk for high levels. For triglycerides, the association with mortality was linear, with higher levels of triglycerides associated with higher mortality risk (Fig. 2). Both LDL-C and high-density lipoprotein cholesterol (HDL-C) showed a U-shaped associated with all-cause and cardiovascular mortality risk (Supplementary Fig. S3 online).

**Figure 3:**
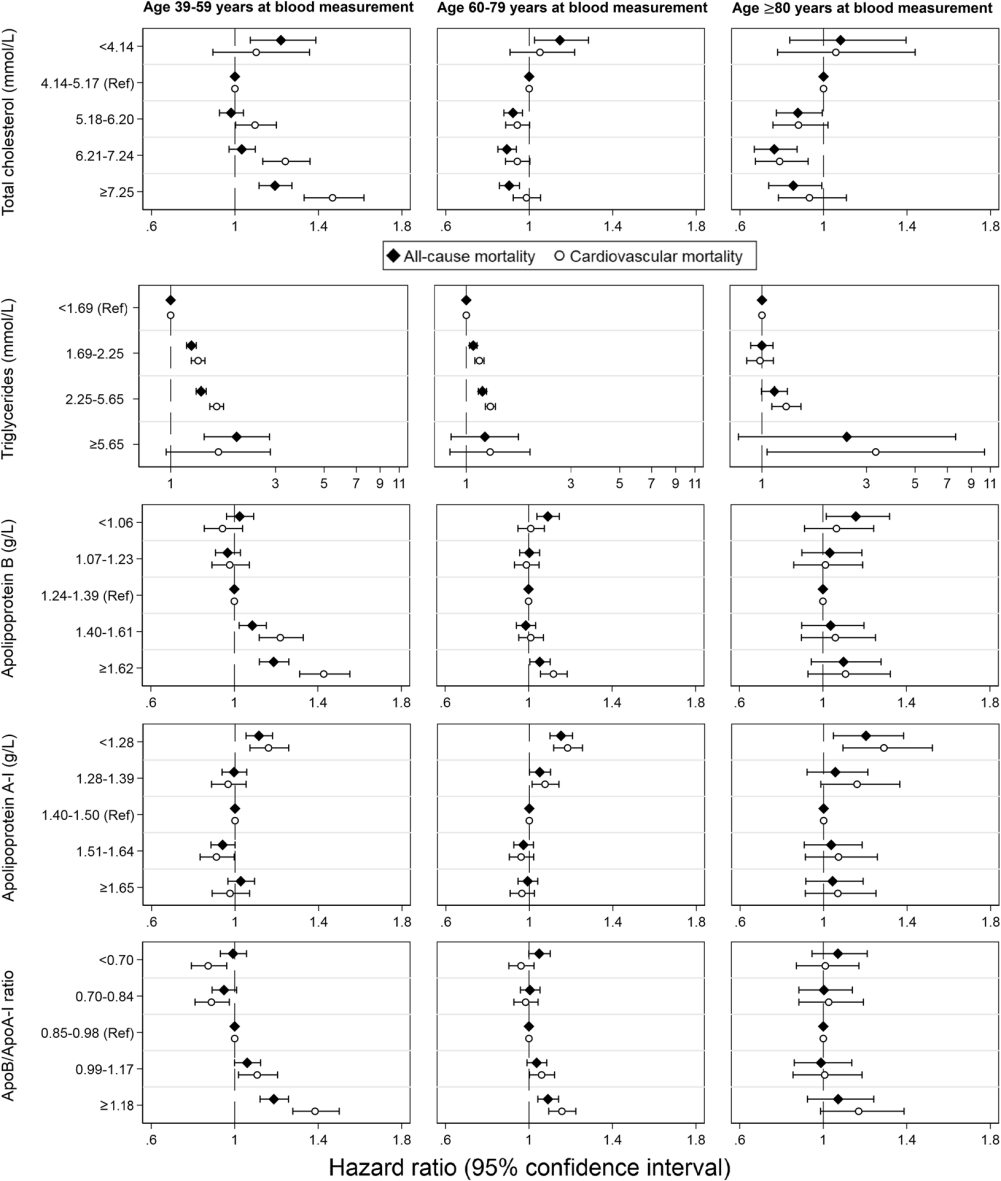
Hazard ratios and 95% confidence interval for all-cause and cardiovascular mortality associated with lipid biomarkers in categories among people aged 39–59 years (left), 60–79 years (middle), and ≥ 80 years (right), respectively, adjusted for age, sex, and history of coronary heart disease, heart failure, atrial fibrillation, hypertension, diabetes, ischemic stroke, and transient ischemic attack.

Figure 3 shows the association between each lipid biomarker in categories and mortality in the three age groups at blood measurement (i.e., 39–59, 60–79 and ≥ 80 years), adjusting for age, sex, and baseline history of CVD. In age group 39–59 years, TC ≥ 7.25 mmol/L was associated with an increased risk of all-cause mortality (HR = 1.19, 95% CI 1.12–1.27) compared to the reference group (4.14–5.17 mmol/L), whereas TC ≥ 7.25 mmol/L in age groups 60–79 and ≥ 80 years was associated with a lower all-cause mortality (HR = 0.90, 95% CI 0.86–0.99 and HR = 0.86, 95% CI 0.74–0.99, respectively). The direction of the association between other lipid biomarkers and mortality was consistent across the three age groups. In all age groups, low ApoA-I (< 1.28 g/L) was associated with increased all-cause and cardiovascular mortality compared to the reference group (1.40–1.50 g/L). An elevated ApoB/ApoA-1 ratio was associated with higher all-cause mortality risk in ages up to 80 years and higher cardiovascular mortality risk in all ages.

Similar to TC, high LDL-C (≥ 4.90 mmol/L) in age group 39–59 years was associated with a higher all-cause mortality compared to the reference group (2.59–3.34 mmol/L), but high LDL-C in age group 60–79 and ≥ 80 years was associated with a slightly lower mortality (Supplementary Fig. S4 online). No substantial age differences were found for the association between HDL-C categories and mortality risk.

## Discussion

In this large population-based study with up to 25 years of follow-up, we had a unique opportunity to identify subgroups of people with distinct lipid profiles and to extend previous knowledge about how lipid profiles and lipid levels at different ages are associated with later life all-cause and cardiovascular mortality. Using cluster analysis, we identified three lipid profiles which revealed that individuals with elevated levels of one lipid often have elevated levels of other lipids, and this was also the cluster that had the strongest association with mortality. We furthermore showed the association of high lipid profile and all-cause and cardiovascular mortality decreased with advancing age and was not apparent anymore in the oldest-old (≥ 80 years at blood measurement). When examining the lipid biomarkers individually, high TC in age group 39–59 years was associated with higher mortality risk but high TC in age group ≥ 80 years was associated with lower mortality risk. On the other hand, high triglycerides, low ApoA-I, and high ApoB/ApoA-I ratio were all associated with higher mortality risk regardless of age at lipid measurement.

Most studies investigating the association between lipids and health outcomes have focused on a selective sample of healthy individuals, which does not capture the heterogeneity of lipid characteristics in the general population. In this study, based on five commonly assessed lipid markers, we found that people with high lipid levels tend to cluster together, and likewise for those with low lipid levels, and such patterns exist in all ages. Individuals in the high lipid profile are characterized by a higher average level of TC, triglycerides, ApoB, and ApoB/ApoA-I ratio and a lower average level of ApoA-I. ApoB and ApoA-I is respectively the major protein component of LDL-C and HDL-C, and ApoB/ApoA-I ratio is an indicator of atherogenic and anti-atherogenic balance in plasma that has been shown to be highly predictive of cardiovascular risk^2,10–12^. Therefore, the high lipid profile identified in our sample represents a lipid phenotype that is highly atherogenic and could potentially lead to the progression of atherosclerotic diseases. This is supported by previous evidence that the combination of high triglycerides and low HDL-C seems to be a common phenotype in middle-aged individuals and is associated with a higher risk of metabolic syndrome and CVD^13,14^. Indeed, we found that people aged 39–59 years belonging to the high lipid profile had a 17% increased risk of all-cause mortality and 36% increased risk of cardiovascular mortality compared to those with the reference lipid cluster, after adjusting for age, sex, and baseline history of CVD. However, high lipid profile among very old people (≥ 80 years) was no longer found to be a risk factor for higher mortality.

When examining the lipid biomarkers individually, we found a U-shaped association for TC and ApoA-I, a J-shaped association for ApoB and AopB/ApoA-I ratio, and a linear association for triglycerides in relation to both all-cause and cardiovascular mortality in the total population. Several recent studies support these findings^8,15–17^. For instance, two recent population-based studies reported a U-shape association between HDL-C and all-cause as well as cardiovascular mortality, with both high and low concentrations associated with higher mortality^8,15^.

In the age-specific analysis, we found that high TC (≥ 7.25 mmol/L) at age 39–59 years was associated with a higher all-cause mortality, but high TC at age 60–79 and ≥ 80 years was associated with a lower mortality. A similar age difference was also found for the association between LDL-C and mortality. Previous evidence on the association between cholesterols and mortality among older adults is conflicting, though most studies support no association or an inverse association^5,6,18–23^. The reason behind such a paradoxical association is not entirely clear. It is possible that the age difference represents selection, so that individuals susceptible to high cholesterols already died before reaching older ages, and those who survived to be included in the study are likely to be less influenced by high cholesterol. Moreover, it has been proposed that in old age TC is a marker of general health, rather than a marker specific for CVD risk^15,16^. However, our results do not suggest that older individuals with higher TC and LDL-C cannot benefit from lipid-lowering treatment. According to the 2021 ESC Guidelines on cardiovascular disease prevention in clinical practice, lipid-lowering treatment is recommended for older adults with a history of CVD in the same way as for younger adults, and in healthy people ≥ 70 years, lipid-lowering treatment shall still be considered if at high risk or above according to specific risk scores^24^. We also found that the lowest TC and LDL-C levels were associated with higher mortality in all the age groups. Cholesterol is important for many body functions including brain metabolism and intracellular transport, and very low cholesterol could increase the susceptibility to cancer, hemorrhagic stroke, or fatal diseases with infectious origins, such as respiratory and gastrointestinal diseases^25–27^.

ApoA-I was suggested to be involved in anti-inflammatory, anti-oxidative, and anti-infectious functions^7^. Data have shown that ApoA-I is more strongly associated with cardiovascular events than HDL-C, suggesting that ApoA-I is a better marker for cardiovascular outcomes than HDL-C^28^. Adding to these findings, our study found that a low ApoA-I is associated with increased all-cause and cardiovascular mortality not only in middle-aged people but also at old age. In addition, we found that an elevated ApoB/ApoA-I ratio is associated with higher all-cause mortality up to 80 years of age and higher cardiovascular mortality in all ages. Previous studies have shown that ApoB/ApoA-I ratio is a better marker for CHD than any other cholesterol indices due to its ability to more accurately measure lipoprotein abnormalities^2,11,12^. Our results indicate although high cholesterols in old age may be associated with lower mortality, low ApoA-I and high ApoB/ApoA-I ratio still retain their ability to confer a higher risk of death, which indicates the clinical relevance of apolipoprotein measures in terms of survival in older individuals. These results are in line with recent findings from randomized controlled trials that not only middle-aged adults but also older individuals (e.g., aged 75 years and older) could benefit from lipid-lowering treatment^29,30^.

The ApoB level represents the total number of atherogenic particles in blood including cholesterol- and triglyceride-containing lipoprotein particles (very-low-density lipoprotein, intermediate-density lipoprotein, and LDL). In subjects with hypertriglyceridemia, type II diabetes, or metabolic syndrome, measurements of LDL-C are commonly normal or even low^2,7,10,31^. This is why physicians too often do not believe that LDL is a risk factor that merits lipid-lowering treatment. However, the small dense LDL particle fraction is the most atherogenic fraction of LDL particles. These are carried by ApoB into the arterial bed; the higher the ApoB value, the higher the risk of CHD. Furthermore, in people with hypertriglyceridemia, type II diabetes, or metabolic syndrome, the protective ApoA-I values are also low, creating a high and “risky” ApoB/ApoA-1 ratio value. Therefore, ApoB measurements are recommended in these subjects in international guidelines^24,32^. Indeed, in the current study high ApoB levels (≥ 1.62 g/L) is associated with higher all-cause and cardiovascular mortality in ages up to 80 years.

Our study has several strengths. First, it is the first study using cluster analysis to identify naturally appearing clusters in the population based on five lipid biomarkers. Second, it has a large sample size representative of the general population and followed for up to 25 years. Death dates and causes of deaths of the participants were retrieved from high quality Swedish national registers with no loss to follow-up. Third, we investigated the entire concentration range of lipid biomarkers, measured at the same clinical laboratory with a consistently applied and well documented methodology, using both splines and clinically meaningful cutoffs. Last, we are one of the few studies that have enough statistical power to perform age-specific analyses including the oldest-old for not only cholesterol and triglyceride but also apolipoprotein levels.

Nevertheless, our study has some limitations. First, due to lack of lifestyle data in the full cohort, we could not further adjust for main cardiovascular risk factors such as smoking, body mass index, and hypertension. Lipid biomarkers analyzed in this study are well-recognized independent risk factors for cardiovascular outcomes, but our results could still be subject to residual confounding due to lack of adjustments for these well-established risk factors. Consequently, the results of this study should be interpreted as descriptive rather than causal associations. Second, we were not able to account for the potential changes in lipid levels over the very long follow-up period. However, changes in lipid levels over time (e.g., as a result of lipid-lowering treatment), would likely have resulted in a dilution of the actual associations between lipids and mortality in the present study. Furthermore, while lipid-lowering drugs such as statins were approved for marketing in Sweden in 1988, which is within the biomarker measurement period (1985–1996), they were not commonly used in the beginning of the 1990s^33^. Therefore, the biomarker levels of most AMORIS participants at the time of blood measurement are most likely untreated values. Yet it is possible that more participants with high cholesterol started lipid-lowering therapies during the follow-up period, which may have tended to reduce the hazard ratios for high cholesterol levels at baseline and subsequent mortality. Finally, the clusters were identified from the Swedish population and may not be directly generalizable to other ethnicities.

Taken together, our findings suggest that, for people who survived to advanced ages, a high cholesterol, triglyceride, and apolipoprotein profile starts to lose some of its ability to confer a higher mortality. High TC at ages younger than 60 was associated with higher mortality, while high TC at older ages was associated with a lower mortality. On the other hand, low ApoA-I was associated with higher all-cause and cardiovascular mortality, regardless of age of lipid measurement, and high ApoB/ApoA-1 ratio was associated with higher all-cause mortality in ages up to 80 and cardiovascular mortality in all ages. Our findings indicate the potential relevance of apolipoprotein measures in terms of survival in both young and older individuals, as well as the importance of age-specific analyses in the evaluation of lipid profiles and health outcomes.

## Methods

### Study population

We used data from the large population-based AMORIS (Apolipoprotein-MOrtality RISk) cohort which includes 812,073 Swedish men and women who submitted their blood samples between 1985 and 1996 for laboratory analysis on a varying number of biomarkers^34^. Blood samples were obtained either through a routine health check-up or outpatient care. All laboratory analyses were performed on fresh blood samples by the Central Automation Laboratory, Stockholm, Sweden, using a consistently implemented and well-documented methodology.

Using the Swedish unique personal identification number, the AMORIS cohort has been linked to a number of national registers, including the National Cause of Death Registry and the Total Population Registry, to provide data on vital status, cause of death, and emigration. All participants were followed from the date of their first laboratory test until the date of death, emigration, or end of follow-up (December 31, 2011), whichever came first. Information on the history of cardiovascular diseases (CVD) at the time of study recruitment was available from the National Patient Registry. A total of 320,105 AMORIS participants aged ≥ 39 years (born between 1889–1945) at the time of blood measurement were eligible for inclusion in the current study. To make the analyses on lipid biomarkers comparable, only participants with complete information on the seven lipid biomarkers from blood measurement were included, resulting in a study sample of 98,270 participants.

This study complies with the Declaration of Helsinki and has been approved by the Regional Ethical committee at Karolinska Institutet, Stockholm, Sweden (reference number 2010/1047-31/1). The Ethical committee has waived the need for informed consent because of the large number of participants in the cohort and because half of them had already died at the time of ethical permits application.

### Assessment of metabolic biomarkers

In the main analyses, TC (mmol/L), triglycerides (mmol/L), ApoB (g/L), and ApoA-I (g/L) were included as lipid biomarkers. The ApoB/ApoA-I ratio was calculated accordingly. Serum levels of TC and triglycerides were measured enzymatically, and ApoA-1 and ApoB were measured by immunoturbidimetric methods. The total coefficient of variation was < 3% for TC and < 5% for other lipid biomarkers. LDL-C and HDL-C levels were calculated based on the levels of TC, triglyceride, and ApoA-I, according to the Jungner method^2,31^, and results for LDL-C and HDL-C are reported in the supplemental materials.

### Ascertainment of vital status and other conditions

Data on dates and causes of death were collected from the Swedish National Cause of Death Registry^35^. The underlying and contributing cause of death was recorded using the International Classification of Disease, Eighth Revision (ICD-8), ICD-9, or ICD-10 codes. In the current study, deaths were grouped as either from any cause (all-cause mortality) or from cardiovascular diseases (ICD-8: 390–458; ICD-9: 390–459; ICD-10: I00-I99). History of CVD at baseline (i.e., coronary heart disease (CHD), heart failure (HF), atrial fibrillation (AF), hypertension, diabetes, ischemic stroke, and transient ischemic attack (TIA)) was identified based on ICD codes through the Swedish Patient Register which includes information on hospital discharges from inpatient care since 1964 regionally and nation-wide since 1987.

### Statistical analysis

Hierarchical cluster analysis (Ward’s method) was used to assess different clustering solutions based on minimum variance sum of squares within clusters^36^. The values of lipid biomarkers were standardized into z-scores and subjects with z-scores above 3SD or below -3SD were considered as outliers and therefore excluded, leaving 94,709 participants in the analyses. The cluster analyses were performed on z-scored age, TC, triglycerides, ApoB, ApoA-I, and ApoB/ApoA-I ratio on a continuous scale. The sequence of mergers in the hierarchical cluster analysis suggested a 3-cluster solution (Online Resource, Fig. S-1). This 3-cluster solution was then used in the *k*-means cluster analysis (i.e., *k* = 3), where the optimal clustering was obtained. We then performed Cox regression models to examine the association between lipid-clusters and all-cause and cardiovascular mortality stratified by age groups at blood measurement, adjusting for age, sex, and baseline history of CVD.

Next, each biomarker was analyzed on a continuous scale in association with all-cause and cardiovascular mortality using Cox regression models with restricted cubic splines. The concentration with the lowest mortality rate was set as reference. Then, we examined each biomarker in categories based on clinical cutoffs for dyslipidemia management^32,37^, and the analyses were stratified by three age groups (i.e., 39–59 years, 60–79 years, and ≥ 80 years at blood measurement). Attained age was used as the time scale, and all models were adjusted for age, sex, and baseline history of CVD. To account for possible reverse causality, individuals that died during the first year of follow-up were censored.

Stata/SE 16.1 (StataCorp LLC, College Station, TX, 77845, USA) and SAS 9.4 for Windows (SAS Institute, Inc. Cary, NC, USA) were used for all analyses.

## Supplementary Information

Supplementary Information.

## Data Availability

The datasets generated during and/or analyzed during the current study are available from the corresponding author on reasonable request.
