# Supplementary material for: The association of apolipoproteins with later-life all-cause and cardiovascular mortality: a population-based study stratified by age
Source: Sci Rep. 2021 Dec 24;11:24440. doi: 10.1038/s41598-021-03959-5 (PMC8709841; doi:10.1038/s41598-021-03959-5)
Supplement: Supplementary file 1 — Supplementary Information. [file 41598_2021_3959_MOESM1_ESM.pdf]

Supplementary materials for:

The association of apolipoproteins with later-life all-cause and cardiovascular mortality: a population-based study stratified by age.

*Mozhu Ding[a], PhD, Alexandra Wennberg[a], PhD, Stina Ek[a], PhD, Giola Santoni[b], PhD, Bruna Gigante[c], MD, PhD, Göran Walldius[a], MD, PhD, Niklas Hammar[a], PhD, Karin Modig[a], PhD

*Corresponding to: Mozhu Ding, Unit of Epidemiology, Institute of Environmental Medicine, Karolinska Institutet, Nobelsväg 13, 17177 Stockholm, Sweden. Tel: +46 08 52480153; Email: mozhu.ding@ki.se

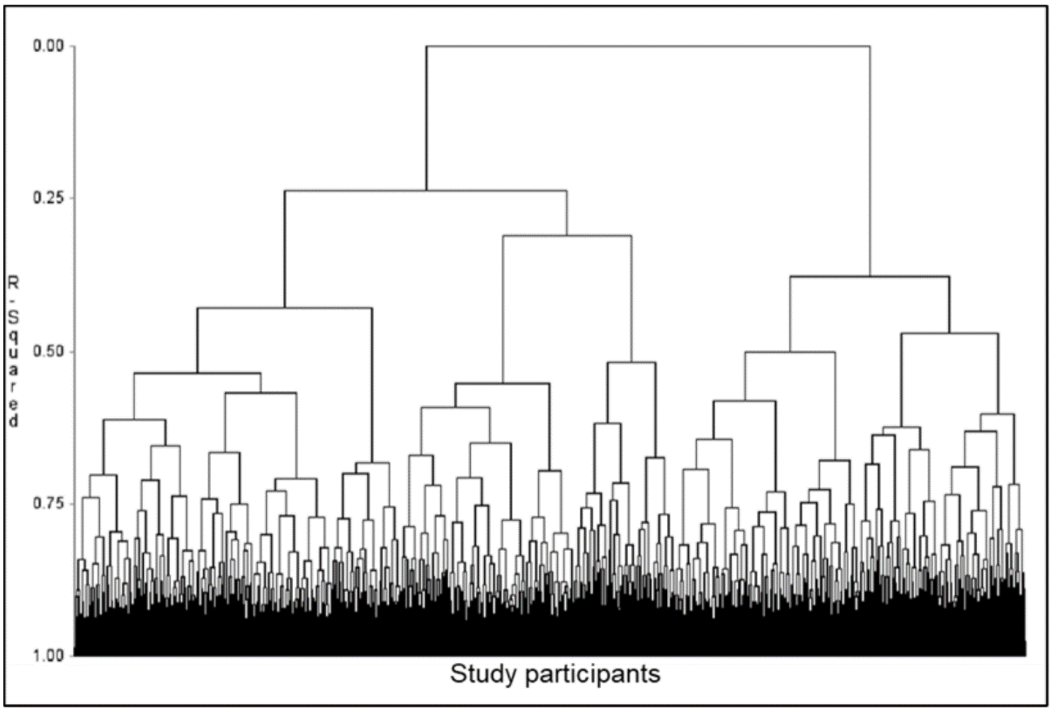

Fig. S1. The sequence of mergers in the dendrogram from hierarchical cluster analysis (using Ward's methods) performed on TC, triglycerides, ApoB, ApoA-I, and ApoB/ApoA-I ratio in z-scores.

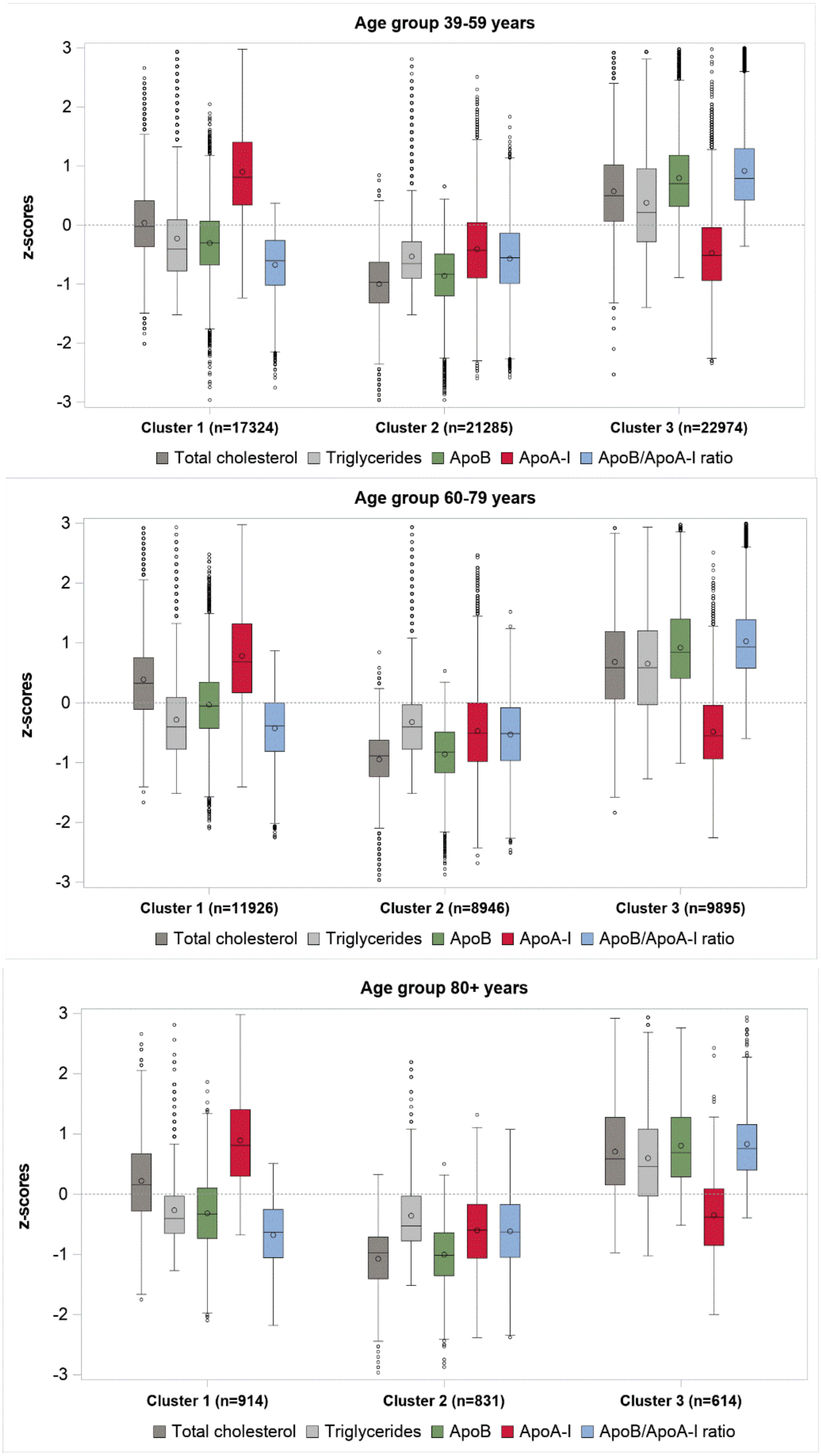

Fig. S2. Boxplots of total cholesterol, triglycerides, ApoB, ApoA-I, and ApoB/ApoA-I ratio in z-scores in the 3 clusters among people aged 39-59, 60-79, and ≥80 years, respectively. Cluster analyses were performed separately in the three age groups.

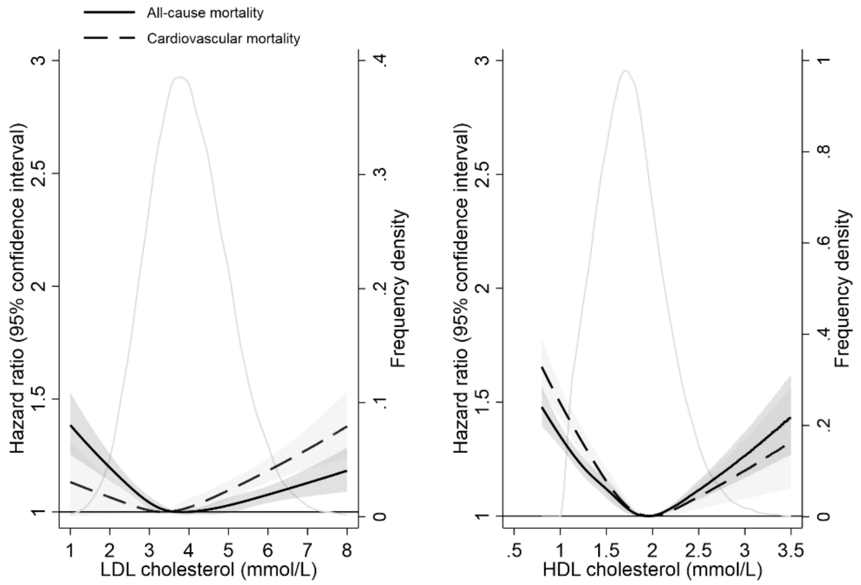

Fig. S3. Hazard ratios and 95% confidence interval for all-cause and cardiovascular mortality associated with concentrations of LDL and HDL cholesterol on a continuous scale over 25 years of follow-up. Hazard ratios (solid line for all-cause mortality and dash line for cardiovascular mortality) and 95% confidence intervals (gray area) are retrieved from Cox regression models with restricted cubic splines, adjusted for age, sex, and history of coronary heart disease, heart failure, atrial fibrillation, hypertension, diabetes, ischemic stroke, and transient ischemic attack. Solid gray line indicates the distribution of lipid biomarkers in the study population.

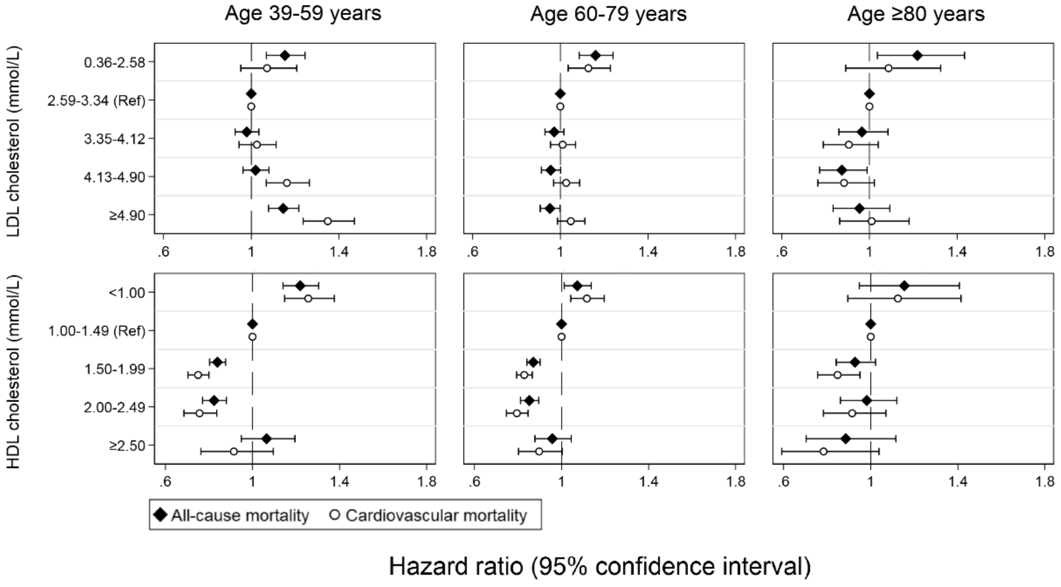

Fig. S4. Hazard ratios and 95% confidence interval for all-cause and cardiovascular mortality associated with LDL and HDL cholesterol in categories among people aged 39-59 years (left), 60-79 years (middle), and ≥80 years (right), respectively, adjusted for age, sex, and history of coronary heart disease, heart failure, atrial fibrillation, hypertension, diabetes, ischemic stroke, and transient ischemic attack.

Table S1. Biomarker concentration levels for the 3 lipid biomarker clusters.

	Cluster 1	Cluster 2	Cluster 3	Kruskal-Wallis test
Number of participants	35375	26124	33210	
Female sex, n (%)	20260 (57.3)	10901 (41.7)	13028 (39.2)	
Age at baseline, mean (SD)	58.4 (10.8)	58.4 (9.4)	57.7 (9.6)	p<0.001
Lipid biomarkers, mean (SD)				
Total cholesterol, mmol/L	6.19 (0.73)	5.03 (0.62)[a]	7.03 (0.84)[a,b]	p<0.001
Triglycerides, mmol/L	1.22 (0.60)	1.05 (0.47)[a]	1.79 (0.72)[a,b]	p<0.001
Apolipoprotein B, g/L	1.23 (0.19)	1.04 (0.17)[a]	1.65 (0.22)[a,b]	p<0.001
Apolipoprotein A-I, g/L	1.63 (0.19)	1.34 (0.16)[a]	1.37 (0.17)[a,b]	p<0.001
ApoB/ApoA-I ratio	0.77 (0.15)	0.79 (0.18)[a]	1.21 (0.18)[a,b]	p<0.001

[a]Statistically different (p<0.001) from cluster 1 for all lipid biomarkers.
[b]Statistically different (p<0.001) from cluster 2 for all lipid biomarkers.
